# Editorial Comment: Phase angle from bioimpedance measurements as a surrogate of cardiovascular disease

**DOI:** 10.1038/s41430-022-01167-6

**Published:** 2022-07-08

**Authors:** Leigh C. Ward

**Affiliations:** School of Chemistry and Molecular Biosciences, The University of Queensland, Brisbane, QLD Australia

**Keywords:** Vascular diseases, Biomarkers

Over the past three decades, bioelectrical impedance analysis (BIA) for the assessment of body composition has gone from a novel application to mainstream clinical use [1]. Originally, single-frequency (50 kHz) BIA used empirically-derived prediction equations to estimate quantitatively total body water, and hence using an assumed hydration coefficient, fat-free mass. Although, generally performing well, it quickly became apparent that prediction equations tended to be population-specific leading to the development and publication of many different equations suitable for use in different populations with attempts being made to develop a “universally” applicable equation, e.g., Kyle et al. [2].

In clinical practice, absolute quantitative data are not always of paramount importance, it may suffice simply to know whether change has occurred, in what direction and whether changes are related to health status or treatment outcome. Untransformed BIA data can be similarly informative. Phase–sensitive BIA devices measure the opposition of body tissues to the flow of an alternating electric current. The measured impedance (Z) comprises two components, resistance (R) and reactance (Xc). Current flow through the body lags behind voltage due to the reactive properties of cell membranes and tissue interfaces acting as imperfect capacitors. This lag effect is expressed as a phase shift or phase angle (PhA, in degrees) and is readily calculated as arctangent (Xc/R) * (180/π). The relationship between PhA and body composition was recognized early (e.g., Baumgartner et al [3].) and has become widely accepted as a prognostic indicator of malnutrition [4]. The clinical utility of PhA has become more widely recognized and extends from its use as a marker of oxidative stress [5] to assessment of athletic health and performance [6].

Cardiovascular disease is one of the leading causes of death world-wide yet its advent and progression are potentially modifiable by behavioral changes such as reducing smoking and alcohol consumption while increasing physical activity [7]. Effective monitoring of at-risk populations is required and PhA has been proposed as an index for predicting CVD, particularly in women [8]. A simple Medline (PubMed) search demonstrates the increasing research activity in this area (Fig. 1).

**Fig. 1:**
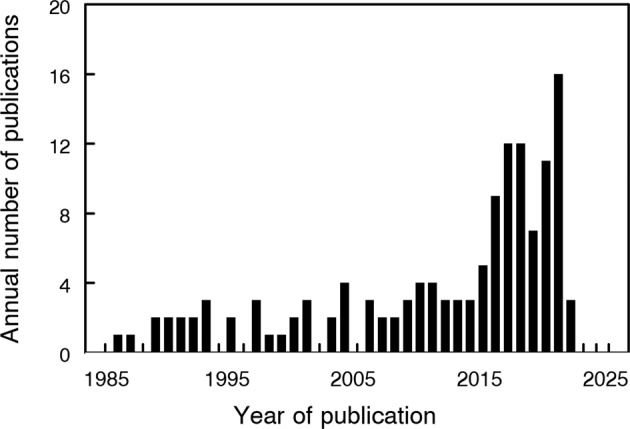
Annual publication rate of papers concerning phase angle and cardiovascular diseases. Data output from Medline (PubMed) search using the following search strategy: (((((((((((((Electric Impedance) OR Impedance, Electric) OR Electrical Impedance) OR Impedance, Electrical) OR Impedance) OR Electric Resistance) OR Resistance, Electric) OR Electrical Resistance) OR Resistance, Electrical) OR Bioelectrical Impedance) OR Impedance, Bioelectrical) OR Bioelectric Impedance) OR Impedance, Bioelectric) AND (Phase Angle) and (Cardiovascular Disease OR Coronary Disease OR CVD).

In this issue of *EJCN*, de Borba et al. provide a systematic review from a meta-analysis of publications assessing the relationship of PhA with cardiovascular disease (CVD) [9]. de Borba et al. identified from their literature search an initial 439 articles that met their selection criteria although screening reduced this to 22 studies (10,010 participants) reviewed and only 8 (2164 participants) for meta-analysis. Overall, findings were that PhA was lower in those with CVD compared to controls irrespective of sex with minimum difference of ~−0.75°. Although this is a small difference, it was noted that technical error of measurement of PhA was much smaller suggesting that differences of this magnitude can be reliably measured. Nevertheless, attention to standardization of BIA measurement and adequate quality control measures should be ensured [4].

The conclusion to be drawn from the meta-analysis of de Borba is that measurement of PhA may be useful in clinical practice when evaluating patients with CVD. It is important to recognize that use of PhA as an index of physiological state is primarily associative. It does not necessarily represent a direct causal link between this electrical parameter and disease state. As pointed out by Lukaski et al., it should not be considered diagnostic but acts as a generic monitor analogous to body temperature as a global measure of health status [4]. Whereas electrical resistance of tissues is due to the inherent resistivity of body water and quantitatively to its volume, both intra- and extracellular, phase angle is generated by tissue reactance. Since reactance is directly related to cell membrane mass and integrity; PhA is in turn considered to represent cell membrane mass and function, frequently combined under the term “cellular health”. Typically, PhA is determined from measurements of whole body (wrist-to-ankle) impedance. It is therefore a representation of overall body tissue cellular health rather than targeted to specific tissues or organs. Consequently, conditions or diseases that exhibit highly focused or localized effects without overall global physiological change may not be amenable to evaluation by PhA. Segmental analysis of body composition using BIA is well established [10] yet segmental PhA remains understudied [11]. Unlike the use of BIA for quantitative body composition assessment, research into the value of PhA in clinical medicine is still relatively immature; it is to be hoped that next few years will see a burgeoning of research activity stimulated in part by publications such as that of de Bora et al. [9].

## Data Availability

Data for Fig. 1 is available on request from the author.
